# Beyond CBD: Inhibitory effects of lesser studied phytocannabinoids on human voltage-gated sodium channels

**DOI:** 10.3389/fphys.2023.1081186

**Published:** 2023-02-20

**Authors:** Carol J. Milligan, Lyndsey L. Anderson, Iain S. McGregor, Jonathon C. Arnold, Steven Petrou

**Affiliations:** ^1^ Florey Institute of Neuroscience and Mental Health, The University of Melbourne, Melbourne, VIC, Australia; ^2^ Brain and Mind Centre, The University of Sydney, Sydney, NSW, Australia; ^3^ Lambert Initiative for Cannabinoid Therapeutics, The University of Sydney, Sydney, NSW, Australia; ^4^ Discipline of Pharmacology, Sydney Pharmacy School, Faculty of Medicine and Health, The University of Sydney, Sydney, NSW, Australia; ^5^ School of Psychology, Faculty of Science, The University of Sydney, Sydney, NSW, Australia; ^6^ Department of Medicine, The University of Melbourne, Melbourne, VIC, Australia

**Keywords:** minor phytocannabinoids, voltage-gated sodium channels, planar patch-clamp electrophysiology, inhibition, potency

## Abstract

**Introduction:** Cannabis contains cannabidiol (CBD), the main non-psychoactive phytocannabinoid, but also many other phytocannabinoids that have therapeutic potential in the treatment of epilepsy. Indeed, the phytocannabinoids cannabigerolic acid (CBGA), cannabidivarinic acid (CBDVA), cannabichromenic acid (CBCA) and cannabichromene (CBC) have recently been shown to have anti-convulsant effects in a mouse model of Dravet syndrome (DS), an intractable form of epilepsy. Recent studies demonstrate that CBD inhibits voltage-gated sodium channel function, however, whether these other anti-convulsant phytocannabinoids affect these classic epilepsy drug-targets is unknown. Voltage-gated sodium (Na_V_) channels play a pivotal role in initiation and propagation of the neuronal action potential and Na_V_1.1, Na_V_1.2, Na_V_1.6 and Na_V_1.7 are associated with the intractable epilepsies and pain conditions.

**Methods:** In this study, using automated-planar patch-clamp technology, we assessed the profile of the phytocannabinoids CBGA, CBDVA, cannabigerol (CBG), CBCA and CBC against these human voltage-gated sodium channels subtypes expressed in mammalian cells and compared the effects to CBD.

**Results:** CBD and CBGA inhibited peak current amplitude in the low micromolar range in a concentration-dependent manner, while CBG, CBCA and CBC revealed only modest inhibition for this subset of sodium channels. CBDVA inhibited Na_V_1.6 peak currents in the low micromolar range in a concentration-dependent fashion, while only exhibiting modest inhibitory effects on Na_V_1.1, Na_V_1.2, and Na_V_1.7 channels. CBD and CBGA non-selectively inhibited all channel subtypes examined, whereas CBDVA was selective for Na_V_1.6. In addition, to better understand the mechanism of this inhibition, we examined the biophysical properties of these channels in the presence of each cannabinoid. CBD reduced Na_V_1.1 and Na_V_1.7 channel availability by modulating the voltage-dependence of steady-state fast inactivation (SSFI, V_0.5_ inact), and for Na_V_1.7 channel conductance was reduced. CBGA also reduced Na_V_1.1 and Na_V_1.7 channel availability by shifting the voltage-dependence of activation (V_0.5_ act) to a more depolarized potential, and for Na_V_1.7 SSFI was shifted to a more hyperpolarized potential. CBDVA reduced channel availability by modifying conductance, SSFI and recovery from SSFI for all four channels, except for Na_V_1.2, where V_0.5_ inact was unaffected.

**Discussion:** Collectively, these data advance our understanding of the molecular actions of lesser studied phytocannabinoids on voltage-gated sodium channel proteins.

## Introduction

Approximately one-third of epilepsy patients worldwide remain resistant to current anti-epileptic drugs (AEDs), generating a critical need for novel anti-convulsant therapies (Kwan et al., 2011). Cannabis-based therapies have potential as novel pharmacotherapies for the treatment of the intractable epilepsies. Phase III clinical trials reported that the phytocannabinoid cannabidiol (CBD) reduced seizures in patients with the intractable epilepsies Dravet syndrome (DS) and Lennox-Gastaut syndrome (LGS) (Devinsky et al., 2017a; Devinsky et al., 2017b; Tang and Fang, 2017; Devinsky et al., 2018; Devinsky et al., 2020; Devinsky et al., 2021).

The introduction of CBD as an approved medicine has generated substantial interest in whether other phytocannabinoids might similarly be developed as novel anti-convulsants. We have recently reported that the lesser studied phytocannabinoids, cannabigerolic acid (CBGA), cannabidivarinic acid (CBDVA), cannabichromenic acid (CBCA) and cannabichromene (CBC) were anti-convulsant in a mouse model of DS (Anderson et al., 2021a; Anderson et al., 2021b; Anderson et al., 2022). However, the mode of action of these compounds remains enigmatic, particularly at epilepsy-relevant drug targets.

Voltage-gated sodium (Na_V_) channels play pivotal roles in controlling central nervous system (CNS) excitability (Catterall, 2014). Pathogenic variants in the main CNS genes *SCN1A*, *SCN2A*, *SCN3A*, and *SCN8A* and the peripheral nervous system (PNS) gene *SCN9A*, that encode the Na_V_ channels Na_V_1.1, Na_V_1.2, Na_V_1.3, Na_V_1.6, and Na_V_1.7, respectively, are associated with well-defined epileptic encephalopathies (Singh et al., 2009; Epi4K, 2013; Mulley et al., 2013; Ademuwagun et al., 2021). In addition, the *SCN4A*, *SCN5A*, and *SCN10A* genes that encode the skeletal muscle Na_V_1.4, the cardiac Na_V_1.5 and the PNS Na_V_1.8 channels, respectively, are associated with other channelopathies (England and de Groot, 2009; Fouda et al., 2022). For example, *SCN4A* mutants cause various neuromuscular disorders (Brugnoni et al., 2022), *SCN5A* mutants are responsible for cardiac syndromes (Verkerk et al., 2018) and pain-related conditions are associated with mutations in *SCN9A* and *SCN10A* (Shen et al., 2022). Therefore, compounds that modify sodium-channel function may have therapeutic efficacy in these various channelopathies. Compounds that inhibit sodium channel function have therapeutic potential for gain-of-function (GOF) mutations, such as those identified in *SCN2A* (Na_V_1.2) and *SCN8A* (Na_V_1.6) in patients with LGS (Epi4K, 2013). Alternatively, compounds that potentiate sodium channel function could prove beneficial for DS, where 80% of patients carry loss-of-function (LOF) mutations in the *SCN1A* gene (Depienne et al., 2009; Richards et al., 2018). The development of Na_V_1.7 inhibitors also hold great promise for the development of novel analgesic agents (Kingwell, 2019).

The phytocannabinoids may potentially yield their anti-seizure and analgesic effects *via* inhibition of Na_V_ channels. We and others have shown that CBD modulates epilepsy-relevant Na_V_ channels (Okada et al., 2005; Ghovanloo et al., 2018; Watkins, 2019; Sait et al., 2020; Milligan et al., 2022). However, the effects of the recently characterized anti-convulsant phytocannabinoids at Na_V_ channels is unknown. The primary aim of the present study was then to explore the Na_V_-dependent pharmacology of five non-psychoactive phytocannabinoids CBGA, CBDVA, CBG, CBCA, and CBC, for four Na_V_ channel isoforms (Na_V_1.1, Na_V_1.2, Na_V_1.6, and Na_V_1.7) associated with epilepsy and pain, and to compare their effects to CBD. All compounds were assessed for their ability to modify sodium channel currents, stably expressed in mammalian cells, using a planar patch-clamp assay.

## Methods

### Tissue culture and transfection

HEK293T cells stably expressing *SCN1A* or *SCN2A* and CHO cells stably expressing *SCN8A* or *SCN9A* were maintained as previously described (Richards et al., 2018; Milligan et al., 2022).

### Phytocannabinoids

The phytocannabinoids were purchased as active pharmaceutical ingredients (APIs) or synthesised with >95% purity. CBD and CBG were purchased from THCPharm, Germany. CBDVA and CBCA were generously provided by Professor Michael Kassiou at the University of Sydney (AUS). CBC was synthesised as previously described (Anderson et al., 2021a). CBGA was provided by Invizyne, United States. All drugs were prepared in 100 mM concentrated stock solutions, in DMSO, and stored at −30°C. Dilutions from these stocks were made each day, in external recording solution, immediately prior to data acquisition. Final drug concentrations contained 0.1% DMSO.

### Planar patch-clamp electrophysiology

Patch-clamp recordings were made using a Patchliner^®^ (Nanion Technologies, Munich, Germany) in the whole-cell configuration as previously described (Richards et al., 2018; Milligan et al., 2022). Briefly, cells were prepared in suspension at a density of 1 × 10^6^-5 × 10^7^ cells/mL. The external recording solution comprised (in mM): 140 NaCl, 4 KCl, 1 MgCl_2_, 2 CaCl_2_, 5 D-glucose, 10 HEPES, pH 7.4 with NaOH, ∼295 mOsm. The internal recording solution comprised (in mM): 50 CsCl, 60 CsF, 10 NaCl, 20 EGTA, 10 HEPES, pH 7.2 with CsOH, ∼285 mOsm. Medium single-hole planar NPC-16 chips with an average resistance of ∼2.5 MΩ were used. Chip and whole-cell capacitance were fully compensated, and 50% series resistance compensation applied. Recordings were acquired at 50 kHz with the low pass filter set to 10 kHz in PATCHMASTER (HEKA Instruments, NY, United States) and performed at 27°C. Offline analysis was performed using Microsoft Excel, MatLab R2019a (MathWorks) and GraphPad Prism 8 (Molecular Devices).

### Voltage clamp protocols

Voltage protocols were used, as previously described (Richards et al., 2018). Briefly, to study the voltage-dependence of activation, cells were held at −120 mV and depolarized to test potentials, in 5 mV increments, between −120 mV and +50 mV for 100 ms. To study steady-state fast inactivation, cells were held at conditioning pre-pulse potentials ranging from −120 mV to +30 mV in 5 mV increments from a holding potential of −120 mV and a test pulse at 5 mV for 20 ms. Recovery from fast inactivation was studied by pre-pulsing the cells to 0 mV from a holding potential of −120 mV for 50 ms, to fully inactivate channels. The voltage was then stepped back to the holding potential for variable interpulse intervals (ipi from 0 to 39 ms in 3 ms increments). To test channel availability, the voltage was stepped to 0 mV for 50 ms.

To determine half-maximal inhibitory concentrations (IC_50_), cells were held at −80 mV, stepped to −120 mV for 200 ms followed by 50 ms test depolarization to 0 mV every 2 s for 30 s in the presence of vehicle control (DMSO). The cells were then exposed to an individual phytocannabinoid (CBD, CBGA, CBDVA, CBG, CBCA or CBC) at concentrations between 0.1 and 100 μM, sequentially for 5 min. Currents for individual cells were averaged over 24 s periods directly before application and following a 5 min exposure of compound. Leak subtraction was applied before normalization of current amplitude. Normalized mean data were fit to the Hill equation.

### Curve fitting and data analysis

To examine the voltage-dependence of activation, normalized current-voltage (*I-V*) relationships were converted to conductance (*G*) using the following equation: *G* = *I*/(*V−V*_*r*_) where V_r_ is the reversal potential for Na^+^. The voltage-dependence of conductance and availability were normalized and fitted to a Boltzmann equation: *G* = 1/(1 + exp [(*V*−*V*_0.5_)/*a*]), where *a* is the slope of the half-maximum, *V* is the potential of the given pulse, and *V*_0.5_ is the potential for the half-maximal activation/inactivation. The time course of inactivation was fitted to a single exponential function *I/Imax* = I_0_+A*exp (-t-t0/τ)+C, where I_0_ is the non-inactivating component, *Imax* is the peak current, t is time, and A is the component for the time constant τ. Time constants were plotted against voltage and the data fitted with a decaying exponential equation *Y* = span*exp (−K*x)+*plateau*, where *span* is the starting point of the curve, *K* is the decay factor, *plateau* is the value the curve decays to, and *x* is time. To measure recovery from inactivation, normalized currents were plotted against ipi and data fitted with equation *I/I*_max_ = 1-exp/(*rc* + x), where *I*_max_ is maximal current; *rc* recovery rate constant; x is time. Peak current (I) was plotted as fractional recovery against the recovery period by normalizing to the maximum current (I_max_) during the conditioning potentials.

### Statistical analyses

All statistical analyses were performed using GraphPad Prism 8 (Molecular Devices) software, with a *p*-value < 0.05 considered statistically significant. One-way ANOVA with Bonferroni correction was applied to consider multiple comparisons. Data values are expressed as mean ± SEM of independent cells.

## Results

**FIGURE 1:**
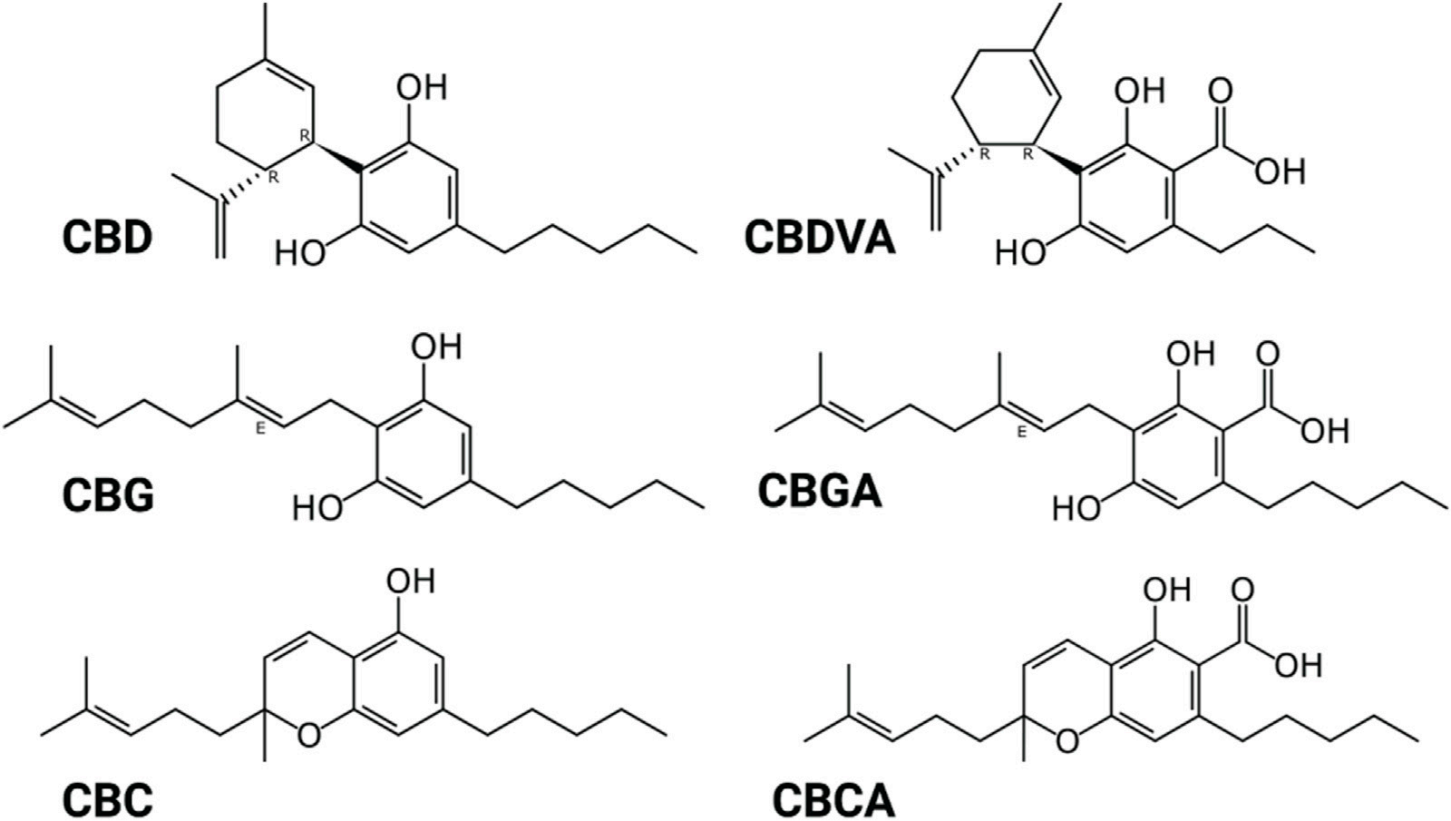
Structure of phytocannabinoids. Created with BioRender.com.

Here, we examined the potency of CBD and the less abundant phytocannabinoids CBGA, CBGVA, CBG, CBCA and CBC on sodium currents of the Na_V_1.1, Na_V_1.2, Na_V_1.6, and Na_V_1.7 channel isoforms expressed in recombinant cells. Figure 1 shows the structure of the phytocannabinoids investigated.

### Potency of CBD for Na_V_ channels

**FIGURE 2:**
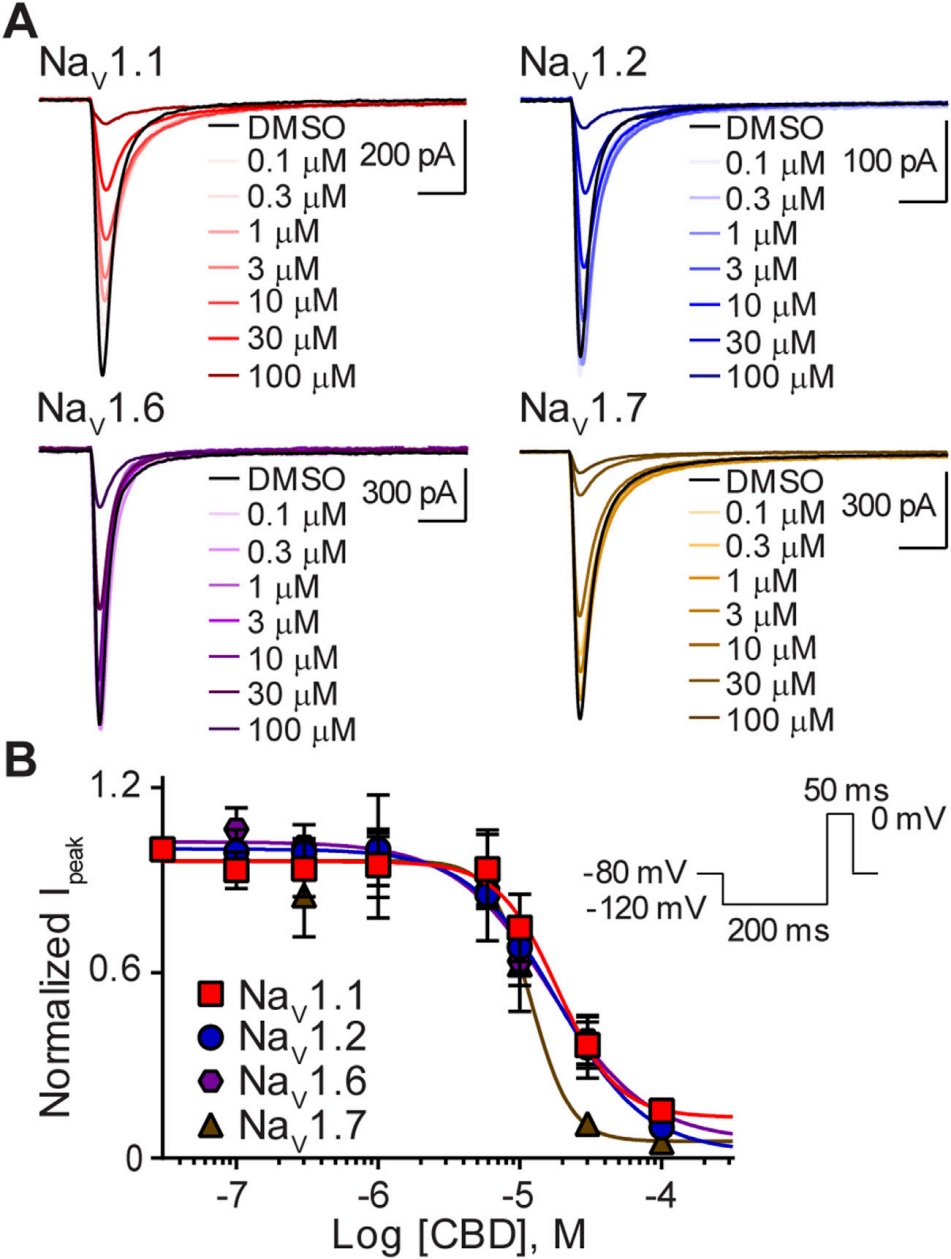
Variable potency of CBD for the Na_V_1.1, Na_V_1.2, Na_V_1.6, and Na_V_1.7 channels. **(A)** Representative current traces for Na_V_1.1, Na_V_1.2, Na_V_1.6 or Na_V_1.7 in the presence of vehicle DMSO (▬) or CBD (0.1–100 μM), as labelled. Horizontal scale bars (2 ms) apply to all traces. **(B)** Potency as a function of CBD concentration (0.1–100 µM) against Na_V_1.1 (*n* = 9), Na_V_1.2 (*n* = 6), Na_V_1.6 (*n* = 7) or Na_V_1.7 (*n* = 7). Data points are mean ± SEM of independent cells. Inset: Schematic of the voltage protocol used to generate these data. *Panel **(B)** reproduced (Milligan et al., 2022). (http://creativecommons.org/licenses/by/4.0/. Original publisher BMC).

Cells expressing a single Na_V_ isoform were used to generate whole-cell current recordings using automated-planar patch-clamp technology. CBD inhibited peak current amplitude of sodium currents, elicited by the four Na_V_ channel subtypes, Na_V_1.1, Na_V_1.2, Na_V_1.6, and Na_V_1.7. Representative current traces at each concentration tested, for each channel subtype, are shown in (Figure 2A). Concentration-response curves were generated for Na_V_1.1, Na_V_1.2, Na_V_1.6, and Na_V_1.7 in cells sequentially exposed to CBD (0.1–100 µM) (Figure 2B). CBD displayed concentration-dependent inhibition of the peak current for all four Na_V_ isoforms tested. Inhibition by CBD was non-selective as its potency, represented by IC_50_ values at each isoform, was not statistically different (Table 1). The steep Hill slopes (Table 1) suggest that CBD’s inhibition is not *via* a one-to-one binding mechanism (Prinz, 2010).

**TABLE 1:** IC_50_ and Hill slope coefficient values for CBD, CBGA and CBDVA on Na_V_1.1, Na_V_1.2, Na_V_1.6 and Na_V_1.7 channels.

	CBD			CBGA			CBDVA		
Isoform	IC_50_ (µM)	Slope	n	IC_50_ (µM)	Slope	n	IC_50_ (µM)	Slope	n
Na_V_1.1	18.5 ± 2.2	2.1 ± 0.8	9	13.6 ± 1.1	2.6 ± 0.5	7	≥50	N.D.	7
Na_V_1.2	18.4 ± 2.6	1.4 ± 0.4	6	14.7 ± 1.1	2.2 ± 0.4	7	≥60	N.D.	8
Na_V_1.6	16.6 ± 1.8	1.3 ± 0.4	7	12.0 ± 1.2	2.3 ± 0.6	6	24.1 ± 1.2	2.1 ± 0.5	6
Na_V_1.7	11.9 ± 2.2	3.1 ± 0.6	7	16.4 ± 1.1	2.5 ± 0.4	8	≥60	N.D.	7

Data points are mean ± SEM, of independent cells; N.D., not determined.

### CBGA inhibited peak sodium currents

**FIGURE 3:**
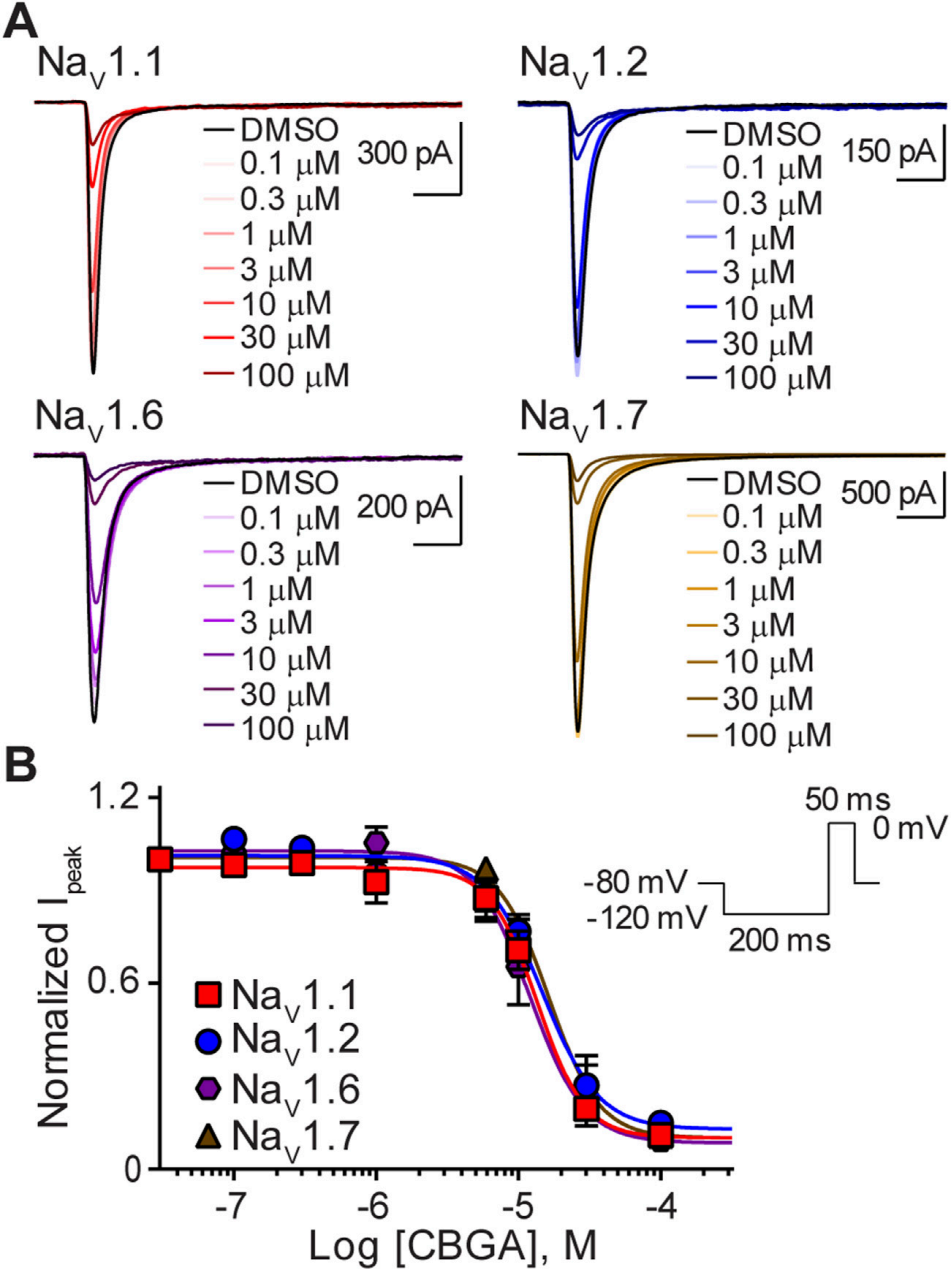
Similar potency of CBGA for the Na_V_1.1, Na_V_1.2, Na_V_1.6 and Na_V_1.7 channels. **(A)** Representative current traces for Na_V_1.1, Na_V_1.2, Na_V_1.6 or Na_V_1.7 in the presence of vehicle DMSO (▬) or CBGA (0.1–100 μM), as labelled. Horizontal scale bars (2 ms) apply to all traces. **(B)** Potency as a function of CBGA concentration (0.1–100 µM) against Na_V_1.1 (*n* = 7), Na_V_1.2 (*n* = 6), Na_V_1.6 (n = 7) or Na_V_1.7 (*n* = 5). Data points are mean ± SEM of independent cells. Inset: Schematic of the voltage protocol used to generate these data.

We next assessed the action of CBGA, the major biosynthetic precursor molecule in *Cannabis sativa*, on sodium channel function. As with CBD, representative current traces at each concentration tested show that CBGA also inhibited the transient sodium currents elicited by Na_V_1.1, Na_V_1.2, Na_V_1.6, and Na_V_1.7 in a concentration-dependent manner (Figures 3A, B). Comparison of calculated IC_50_ values across isoforms, shows that CBGA was also a non-selective inhibitor with comparable potencies to CBD. The Hill coefficients being greater than one are suggestive of CBGA having more than one binding site (Table 1) (Prinz, 2010).

### CBDVA selectively inhibited Na_V_1.6 currents

**FIGURE 4:**
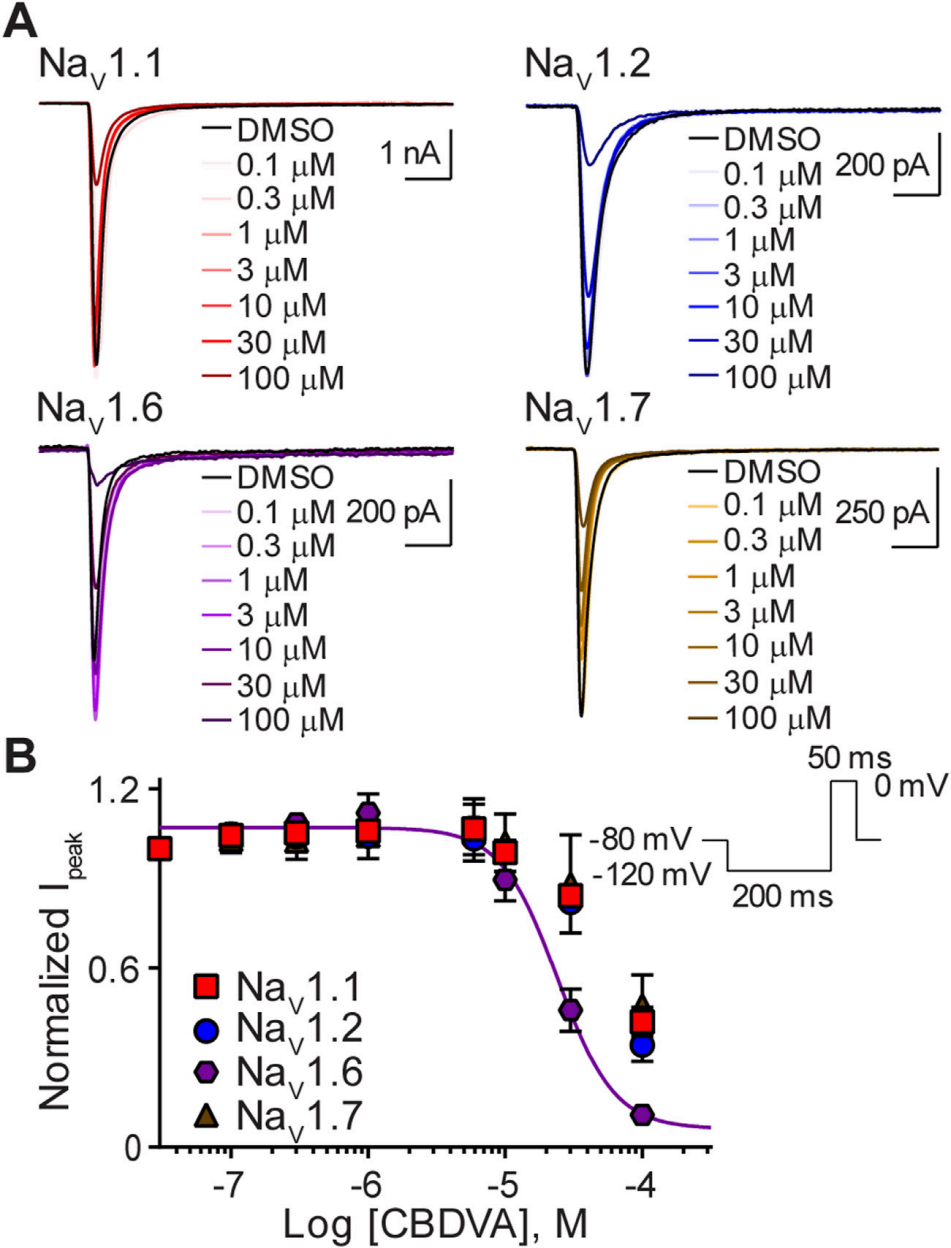
Effect of CBDVA on the Na_V_1.1, Na_V_1.2, Na_V_1.6, and Na_V_1.7 channels. **(A)** Representative current traces for Na_V_1.1, Na_V_1.2, Na_V_1.6 or Na_V_1.7 in the presence of vehicle DMSO (▬) or CBDVA (0.1–100 μM), as labelled. Horizontal scale bars (2 ms) apply to all traces. **(B)** Potency as a function of CBDVA concentration (0.1–100 µM) against Na_V_1.1 (*n* = 7), Na_V_1.2 (*n* = 8), Na_V_1.6 (*n* = 6) or Na_V_1.7 (*n* = 7). Data points are mean ± SEM of independent cells. Inset: Schematic of the voltage protocol used to generate these data.

Next, we sought to determine the effects of CBDVA on this subset of sodium channels. Representative traces illustrate that, like CBD and CBGA, CBDVA also inhibited peak currents of the Na_V_1.1, Na_V_1.2, Na_V_1.6, and Na_V_1.7 channels, however, at the highest concentration examined (100 µM) maximal inhibition of Na_V_1.1, Na_V_1.2 and Na_V_1.7 currents was not observed (Figure 4A). Na_V_1.6 currents were selectively inhibited by CBDVA (0.1–100 μM) in a concentration-dependent manner (Figure 4B), yielding an IC_50_ value in the low micromolar range (Table 1). Because CBDVA (100 μM) only partially inhibited currents elicited by Na_V_1.1, Na_V_1.2, and Na_V_1.7, we were unable to calculate IC_50_ values, and thus, Hill slope coefficients (Table 1).

### Differential effects of the minor phytocannabinoids CBG, CBCA, and CBC

**FIGURE 5:**
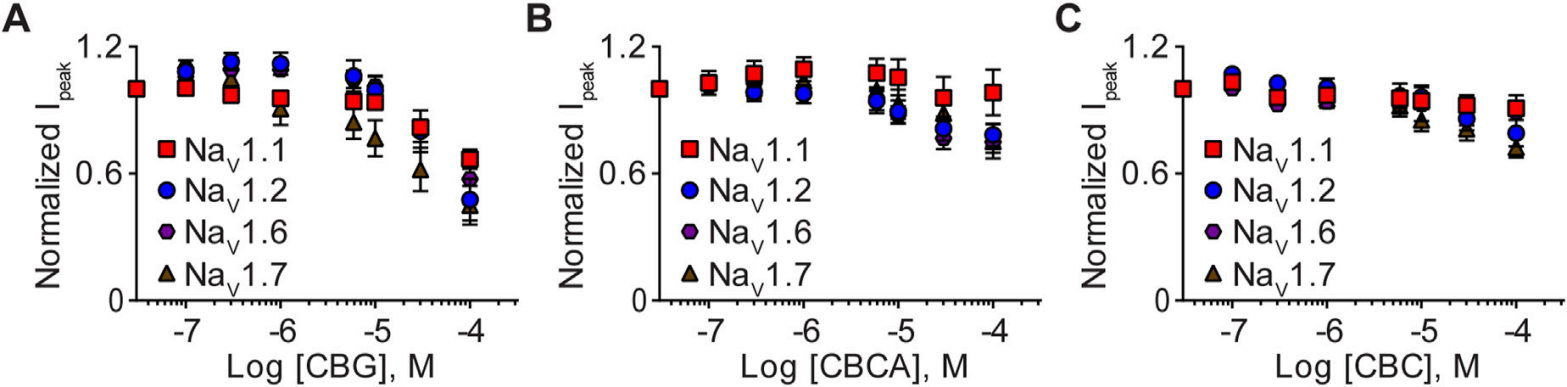
Effects of CBG, CBCA, and CBC on Na_V_1.1, Na_V_1.2, Na_V_1.6 and Na_V_1.7 peak currents. Normalized mean concentration-response curves for **(A)** CBG (0.1–100 µM) against Na_V_1.1 (*n* = 7), Na_V_1.2 (*n* = 8), Na_V_1.6 (*n* = 7) or Na_V_1.7 (*n* = 7); **(B)** CBCA (0.1–100 µM) against Na_V_1.1 (*n* = 8), Na_V_1.2 (*n* = 6), Na_V_1.6 (*n* = 10) or Na_V_1.7 (*n* = 10); and **(C)** CBC (0.1–100 µM) against Na_V_1.1 (*n* = 7), Na_V_1.2 (*n* = 9), Na_V_1.6 (*n* = 7) or Na_V_1.7 (*n* = 11). Data points are mean ± SEM of independent cells.

Finally, we examined the effects of CBG, CBCA, and CBC (0.1–100 μM) on Na_V_1.1, Na_V_1.2, Na_V_1.6, and Na_V_1.7 channel function. Concentration-response curves demonstrate that CBG, CBCA, and CBC modestly inhibited sodium currents, suggesting that the channels are less sensitive to these minor phytocannabinoids (Figure 5). Given the modest inhibition and that 100 µM concentrations did not cause maximal inhibition, IC_50_ values and thus Hill slope coefficients were not determined.

### The effects of CBD on the biophysical properties of Na_V_1.1, Na_V_1.2, Na_V_1.6, and Na_V_1.7

**FIGURE 6:**
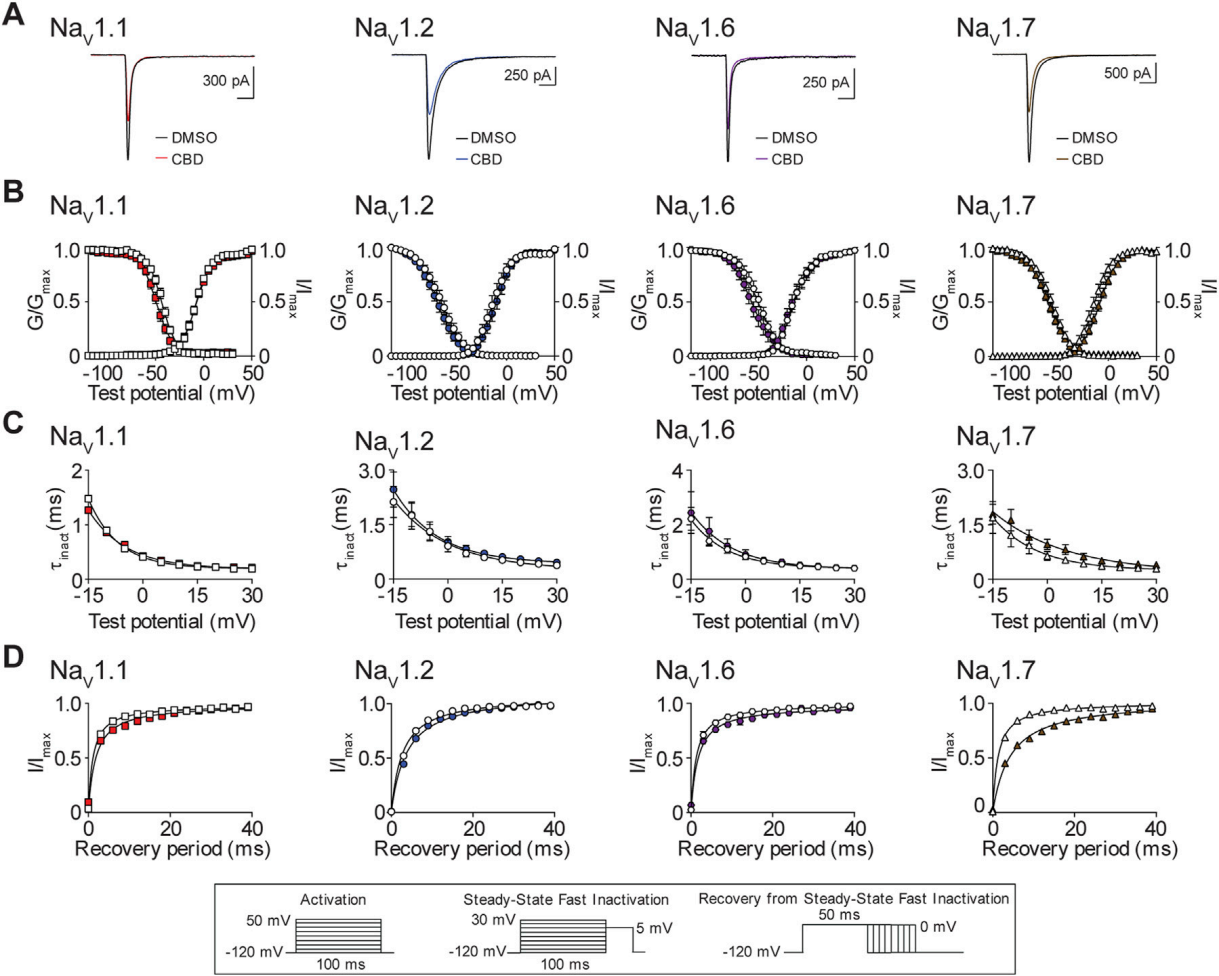
Biophysical effects of CBD on the Na_V_1.1, Na_V_1.2, Na_V_1.6 and Na_V_1.7 channels. **(A)** Representative current traces in the presence of vehicle DMSO (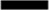) or IC_50_ concentration of CBD for each channel (Na_V_1.1: 18.5 μM 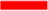; Na_V_1.2: 18.4 μM 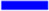; Na_V_1.6: 16.6 μM 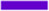; Na_V_1.7: 11.9 μM 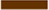). **(B)** Voltage-dependence of normalized peak conductance (G/G_max_) and SSFI (I/I_max_) in the presence of vehicle DMSO (open symbol) or IC_50_ concentration of CBD for Na_V_1.1 (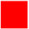; *n* = 20), Na_V_1.2 (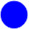; *n* = 12), Na_V_1.6 (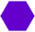; *n* = 17), or Na_V_1.7 (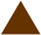; *n* = 17). Boltzmann curves were fitted to pooled averages of peak conductance. **(C)** Time constant of steady-state fast inactivation (τ_inact_), as a function of voltage, in the presence of DMSO vehicle (open symbols) or IC_50_ concentration of CBD for each channel (closed symbols). **(D)** Recovery of channel availability from fast inactivation as a function of time, in the presence of DMSO vehicle (open symbols) or IC_50_ concentration of CBD (closed symbols) for each channel. Data points are mean ± SEM of independent cells. Inset: Schematics of the voltage protocols used to generate data for Figures 6–8.

Next, we examined the effects of the IC_50_ concentration of CBD for the Na_V_1.1, Na_V_1.2, Na_V_1.6, and Na_V_1.7 channels (Table 1), on the biophysical properties of activation, steady-state fast inactivation (SSFI) and recovery from SSFI. We show representative current traces before and after exposure to CBD for each channel subtype (Figure 6A). Peak channel conductance shows that CBD did not alter the midpoint of activation, for the Na_V_1.1, Na_V_1.2, and Na_V_1.6 channels, when compared to vehicle DMSO. However, CBD did induce a significant depolarizing shift in the conductance curve of the Na_V_1.7 channel, which is consistent with a decrease in channel availability. In addition, CBD significantly affected the apparent valence (slope, *a*) of activation for Na_V_1.1, Na_V_1.2, and Na_V_1.7, but not Na_V_1.6. Although there is no effect on the voltage-dependence of activation for Na_V_1.1 and Na_V_1.2, an increase in the slope of the conductance curves was observed. An increase in the slope factor suggests that CBD has an enhancing effect on these three channels, since a larger slope factor indicates greater activation of the channel at voltages negative to the half-activation voltage. For Na_V_1.7, although depolarizing the conductance curve and increasing the slope of the conductance produce opposing effects, the overall effect is inhibitory (Figure 6B; Table 2). We also measured the effects of the IC_50_ concentration of CBD on the voltage dependence of SSFI for each channel. CBD caused a hyperpolarizing shift in mid-point of SSFI for Na_V_1.1 and Na_V_1.7, which is indicative of a reduction in channel availability as the channels have a greater tendency to move into the inactivated state. For Na_V_1.7, this shift was accompanied by an increase in the slope of inactivation. The time constant of fast inactivation, compared at +5mV, for Na_V_1.7 was significantly increased by CBD, indicating a slowing of inactivation, which is consistent with reduced function. Despite CBD causing a shift in the voltage-dependence of inactivation for Na_V_1.1, the time constant of inactivation was unaffected. No significant changes in SSFI were observed with Na_V_1.2 or Na_V_1.6 (Figure 6C; Table 2). Recovery from SSFI was significantly slower for Na_V_1.1, Na_V_1.2, Na_V_1.6, and Na_V_1.7, in the presence of CBD, suggestive of reduced channel availability which is consistent with a decrease in channel activity (Figure 6D; Table 2).

**TABLE 2:** Change in the biophysical properties of activation, inactivation, and recovery from steady-state fast inactivation of Na_V_1.1, Na_V_1.2, Na_V_1.6, and Na_V_1.7 isoforms following application of IC_50_ concentrations of CBD, CBGA, and CBDVA.

	Activation		Inactivation			Recovery	
Isoform-compound	△ V_0.5_ act (mV)	△ Slope factor	△ V_0.5_ inact (mV)	△ slope factor	△ τ_inact_ SSFI at 5 mV	△ *rc*	n
Na_V_1.1—CBD	0.3 ± 1.2	0.7 ± 0.2**	−5.6 ± 1.1****	0.5 ± 0.3	0.03 ± 0.02	0.9 ± 0.3**	20
Na_V_1.2—CBD	3.3 ± 2.7	1.0 ± 0.3*	−4.2 ± 1.9	0.1 ± 0.2	0.08 ± 0.07	0.9 ± 0.3*	12
Na_V_1.6—CBD	−0.6 ± 1.1	0.2 ± 0.2	−5.3 ± 3.6	−0.3 ± 0.7	0.03 ± 0.06	0.3 ± 0.1*	17
Na_V_1.7—CBD	5.7 ± 2.3*	1.9 ± 0.3****	−3.7 ± 1.2**	1.2 ± 0.3***	0.3 ± 0.1*	3.6 ± 0.6****	20
Na_V_1.1—CBGA	3.9 ± 1.2**	3.1 ± 1.7	1.0 ± 1.29	0.2 ± 0.8	0.4 ± 0.1*	0.4 ± 0.1**	11
Na_V_1.2—CBGA	1.8 ± 2.3	1.9 ± 1.0	−3.0 ± 1.5	2.5 ± 0.8**	0.08 ± 0.1	1.4 ± 0.5**	16
Na_V_1.6—CBGA	0.1 ± 1.6	−0.4 ± 0.3	−0.5 ± 2.4	1.1 ± 0.2**	0.2 ± 0.2	0.9 ± 0.4	7
Na_V_1.7—CBGA	4.6 ± 1.7*	1.1 ± 0.2***	−5.5 ± 2.1*	1.3 ± 0.5*	0.3 ± 0.06***	3.2 ± 0.8***	22
Na_V_1.1—CBDVA	13.0 ± 1.6****	1.7 ± 0.4***	−10.1 ± 2.3**	1.8 ± 0.3***	0.4 ± 0.05****	1.0 ± 0.3**	12
Na_V_1.2—CBDVA	11.5 ± 1.5****	0.9 ± 0.2****	−2.3 ± 1.5	1.0 ± 0.2***	0.4 ± 0.06****	0.7 ± 0.2**	18
Na_V_1.6—CBDVA	10.4 ± 2.9**	1.9 ± 0.7*	−3.0 ± 1.3*	0.5 ± 0.3	0.3 ± 0.09**	0.6 ± 0.1***	20
Na_V_1.7—CBDVA	8.7 ± 1.8***	0.9 ± 0.2***	−4.1 ± 0.9***	1.6 ± 0.3****	0.3 ± 0.07**	1.6 ± 0.4***	23

△, Change; V_0.5_ act/inact, voltage-dependence of half-activation or -inactivation; τ_inact_, time constant; SSFI, steady-state fast inactivation; *rc*, recovery rate constant. Data points are mean ± SEM, of independent cells. Statistical significance is marked as *p < 0.05, **p < 0.01, ***p < 0.001, ****p < 0.0001. Statistical comparisons were made with paired Student’s t-test.

### The effects of CBGA on the biophysical properties of Na_V_1.1, Na_V_1.2, Na_V_1.6, and Na_V_1.7

**FIGURE 7:**
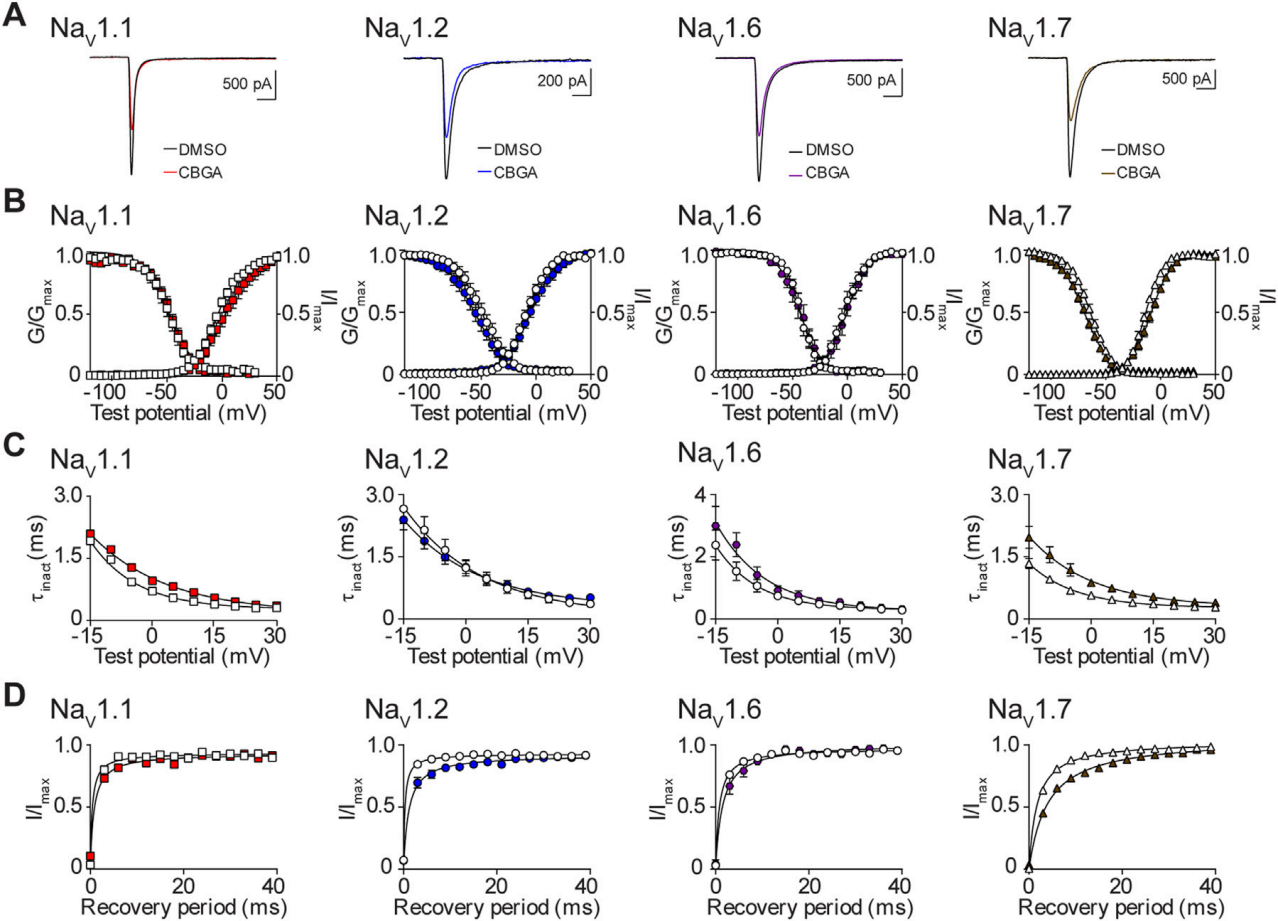
Biophysical effects of CBGA on the Na_V_1.1, Na_V_1.2, Na_V_1.6 and Na_V_1.7 channels. **(A)** Representative current traces in the presence of vehicle DMSO (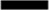) or IC_50_ concentrations of CBGA for each channel (Na_V_1.1: 13.6 μM 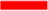; Na_V_1.2: 14.7 μM 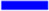; Na_V_1.6: 12.0 μM 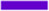; Na_V_1.7: 16.4 μM 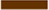). **(B)** Voltage-dependence of normalized peak conductance (G/G_max_) and SSFI (I/I_max_) in the presence of vehicle DMSO (open symbol) or IC_50_ concentration of CBGA for Na_V_1.1 (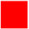; *n* = 11), Na_V_1.2 (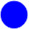; *n* = 16), Na_V_1.6 (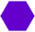; *n* = 7), or Na_V_1.7 (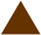; *n* = 22). Boltzmann curves were fitted to pooled averages of peak conductance. **(C)** Time constant of steady-state fast inactivation (τ_inact_), as a function of voltage, in the presence of DMSO vehicle (open symbols) or IC_50_ concentration of CBGA for each channel (closed symbols). **(D)** Recovery of channel availability from fast inactivation as a function of time, in the presence of DMSO vehicle (open symbols) or IC_50_ concentration of CBGA (closed symbols) for each channel. Data points are mean ± SEM of independent cells.

Next, we examined the effects of the IC_50_ concentration of CBGA for Na_V_1.1, Na_V_1.2, Na_V_1.6, and Na_V_1.7 (Table 1) on channel biophysics. We show representative current traces in the presence of DMSO and after exposure to CBGA for each isoform (Figure 7A). CBGA induced significant depolarizing shifts in the voltage-dependence of activation for the Na_V_1.1 and Na_V_1.7 channels. However, CBGA did not affect the mid-point of conductance for Na_V_1.2 or Na_V_1.6. For Na_V_1.7, CBGA also caused a significant enhancement of the slope of the activation curve, an effect that was not observed for the other three channels (Figure 7B; Table 2). Examination of the effects of CBGA on SSFI, revealed a negative shift in the voltage-dependence for Na_V_1.7, accompanied by an increase in the value of the slope factor. However, CBGA had no effect on the voltage-dependence of inactivation for Na_V_1.1, Na_V_1.2 or Na_V_1.6, although slope factor values were increased. In addition, CBGA caused a slowing of the time course of inactivation for Na_V_1.1 and Na_V_1.7 (Figure 7C; Table 2). Recovery from SSFI was significantly slower for Na_V_1.1, Na_V_1.2 and Na_V_1.7, but not Na_V_1.6, in the presence of CBGA at each isoform (Figure 7D; Table 2).

### The effects of CBDVA on the biophysical properties of Na_V_1.1, Na_V_1.2, Na_V_1.6, and Na_V_1.7

**FIGURE 8:**
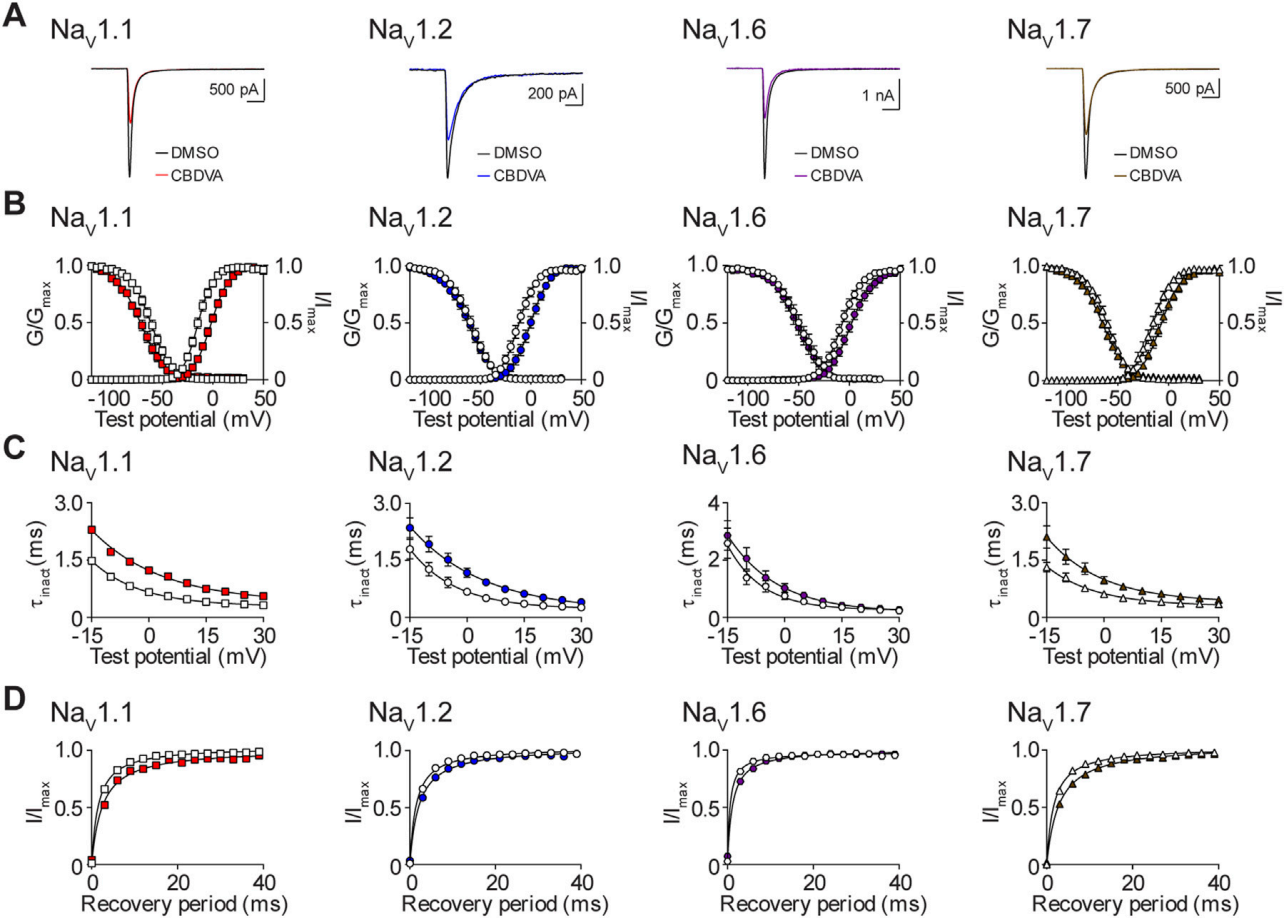
Biophysical effects of CBDVA on the Na_V_1.1, Na_V_1.2, Na_V_1.6 and Na_V_1.7 channels. **(A)** Representative current traces in the presence of vehicle DMSO (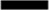) or IC_50_ concentrations of CBDVA for each channel (Na_V_1.1: 50 μM 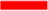; Na_V_1.2: 60 μM 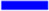; Na_V_1.6: 24.1 μM 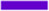; Na_V_1.7: 60 μM 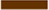). **(B)** Voltage-dependence of normalized peak conductance (G/G_max_) and SSFI (I/I_max_) in the presence of vehicle DMSO (open symbol) or IC_50_ concentration of CBDVA for Na_V_1.1 (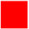; *n* = 12), Na_V_1.2 (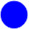; *n* = 18), Na_V_1.6 (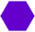; *n* = 11), or Na_V_1.7 (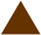; *n* = 23). Boltzmann curves were fitted to pooled averages of peak conductance. **(C)** Time constant of steady-state fast inactivation (τ_inact_), as a function of voltage, in the presence of DMSO vehicle (open symbols) or IC_50_ concentration of CBDVA for each channel (closed symbols). **(D)** Recovery of channel availability from fast inactivation as a function of time, in the presence of DMSO vehicle (open symbols) or IC_50_ concentration of CBDVA (closed symbols) for each channel. Data points are mean ± SEM of independent cells.

Finally, we assessed the effects of CBDVA, at the IC_50_ concentration for Na_V_1.1, Na_V_1.2, Na_V_1.6 and Na_V_1.7 (Table 1), on the biophysical properties of channel function. Representative current traces for vehicle control and CBDVA for each subtype are shown (Figure 8A). CBDVA induced robust depolarizing shifts in the voltage-dependence of activation for Na_V_1.1, Na_V_1.2, Na_V_1.6, and Na_V_1.7, together with increases in the slope of the conductance curves, when compared to DMSO (Figure 8B; Table 2). Examination of the effects of CBDVA on SSFI, revealed a hyperpolarizing shift in the mid-point of inactivation for Na_V_1.1, Na_V_1.6, and Na_V_1.7. For Na_V_1.1 and Na_V_1.7, this negative shift was accompanied by an increase in the slope factor. In contrast, CBDVA had no effect on the inactivation curve for Na_V_1.2, although it did cause an increase in the slope factor. All four channels had slower inactivation time courses in the presence of CBDVA (Figure 8C; Table 2). CBDVA also slowed the recovery from SSFI for Na_V_1.1, Na_V_1.2, Na_V_1.6, and Na_V_1.7 (Figure 8D; Table 2).

## Discussion

CBD is now a well-established anti-convulsant used to treat the intractable epilepsies (Devinsky et al., 2016; Pisanti et al., 2017). This has inspired research addressing whether other less well characterized phytocannabinoids might similarly have anti-seizure properties. Indeed, recent studies have shown that several minor cannabinoids have anti-seizure effects in mouse models including CBGA, CBDVA, CBCA, and CBC (Anderson et al., 2019b; Anderson et al., 2021a; Anderson et al., 2021b; Anderson et al., 2022; Benson et al., 2022). However, the molecular mode of action of these compounds is poorly understood. Here we advance the molecular characterization of the minor phytocannabinoids by assessing their effects at voltage-gated sodium channels. Moreover, we compared the potency of these compounds to those of CBD, which we have recently reported under the same experimental conditions using planar patch-clamp electrophysiology (Milligan et al., 2022).

CBD and CBGA inhibited peak current amplitude of a subset of sodium channel isoforms expressed in recombinant mammalian cells. Both compounds produced comparable, non-selective inhibition of Na_V_1.1, Na_V_1.2, Na_V_1.6, and Na_V_1.7 with IC_50_ values in the low micromolar range. In contrast, CBDVA selectively inhibited the Na_V_1.6 channel, again in the low micromolar range, and displayed lower potency for Na_V_1.1, Na_V_1.2, and Na_V_1.7. Interestingly, the inhibition of sodium currents, by CBD, CBGA, and CBDVA, have steep Hill slopes which suggests that their inhibition is not *via* a one-to-one binding mechanism (Prinz, 2010). The other phytocannabinoids tested CBG, CBCA, and CBC only partially inhibited Na_V_1.1, Na_V_1.2, Na_V_1.6, and Na_V_1.7 channel currents with 100 µM concentrations unable to produce maximal inhibition.

To better understand the mechanism by which CBD, CBGA, and CBDVA inhibit sodium currents, we examined the impact of the IC_50_ concentration of each compound on the biophysical properties of the Na_V_1.1, Na_V_1.2, Na_V_1.6, and Na_V_1.7 channels. We found that CBD decreased the tendency of Na_V_1.1 and Na_V_1.7 to move into the inactivated state, thus reducing channel availability, an effect previously reported for the Na_V_1.1 channel (Ghovanloo et al., 2018). In addition, CBD shifted the voltage-dependence of activation to a more depolarized potential and slowed the kinetics of inactivation of Na_V_1.7 further reducing channel availability. Moreover, CBD slowed the rate of recovery from SSFI of all four Na_V_ channels, an effect consistent with functional inhibition. Similarly, CBGA reduced Na_V_1.1 and Na_V_1.7 channel availability by modifying the voltage-dependence of activation, slowing recovery from SSFI, and slowing the time course of fast inactivation. In addition, CBGA disrupted the SSFI of Na_V_1.7 and slowed recovery from inactivation of the Na_V_1.2 channel. CBDVA reduced channel availability by modifying conductance, SSFI and recovery from SSFI for all four channels, except for Na_V_1.2, where V_0.5_ inact was not affected. Anti-seizure medications that inhibit sodium channels are contraindicated for the treatment of DS (Wirrell et al., 2017; de Lange et al., 2018). Despite this, CBD, which has been shown by us and others to inhibit Na_V_1.1 currents, *in vitro* (Ghovanloo et al., 2018; Milligan et al., 2022), reduces seizure frequency in this group of patients. The inhibition of Na_V_1.1, by CBD and CBGA, demonstrated here, suggest that these phytocannabinoids may also be promising therapeutics for patients who carry a GOF recurrent missense variant (p.Thr226Met) in the *SCN1A* gene, which presents with an extremely severe developmental and early infantile epileptic encephalopathy phenotype (Berecki et al., 2019). As CBD and CBGA also inhibit Na_V_1.2, they could have therapeutic potential in LGS patients with *SCN2A* GOF mutations (Epi4K, 2013).

Na_V_1.6 also presents an interesting therapeutic target for CBD, CBGA, and CBDVA, because inhibition of Na_V_1.6 reduces epileptiform events in a zebrafish model of DS, providing a neuronal counterbalance to the haploinsufficiency of the *Scn1a* model (Weuring et al., 2020). This could be particularly relevant for CBDVA, which in our hands selectively inhibits Na_V_1.6 channel currents. In addition to this, we have previously demonstrated that CBGA and CBDVA have anti-convulsant properties against thermally induced seizures in a *Scn1a* ^+/−^ mouse model of DS (Anderson et al., 2021a; Anderson et al., 2021b), suggesting that inhibition of Na_V_1.2 and Na_V_1.6 channels could also be compensating for the haploinsufficiency in our DS model. However, if you compare the estimated brain CBGA and CBDVA concentrations attained at anti-convulsant doses (CBGA: 720 nM–4 µM, CBDVA: 5.5 µM) to the IC_50_ values determined here (CBGA: 12–16.4 µM, CBDVA: 24.1 µM), it seems unlikely that Na_V_ inhibition contributes to the anti-convulsant efficacy of CBGA and CBDVA against hyperthermia-induced seizures (Anderson et al., 2019b). Caution should be taken when considering CBGA as a potential therapeutic because we reported proconvulsive effects when CBGA was used as a monotherapy on spontaneous seizures in the same DS mouse model and in the 6-Hz acute seizure model (Anderson et al., 2021b).

CBG, one of the major constituents of *Cannabis sativa* (Nachnani et al., 2021), has previously been shown to inhibit sodium channel currents *in vitro*, however, it was ineffective as an anti-convulsant in a PTZ-induced acute seizure model (Hill et al., 2014). Moreover, it was ineffective against hyperthermia-induced seizures in a *Scn1a* ^+/−^ mouse model of DS (Anderson et al., 2021b). In our hands, CBG produces modest inhibitory effects on peak currents elicited by this subset of sodium channels. This differs slightly from previous reports showing CBG to act as a low affinity inhibitor of sodium channels (IC_50_ ∼2–22 μM) (Hill et al., 2014; Ghovanloo et al., 2022). Different voltage protocols or model systems were used in these studies; however, this seems an unlikely explanation for the discrepancy.

In an early study, CBC was found to be ineffective in an electrically induced seizure model (Davis and Hatoum, 1983). However, more recently we showed both CBC and CBCA displayed anti-convulsant properties against hyperthermia-induced seizures in *Scn1a* ^+/−^ mice (Anderson et al., 2021a). Here we found that CBC and CBCA displayed very limited inhibition of Na_V_1.1, Na_V_1.2, Na_V_1.6, and Na_V_1.7 channels, suggesting that the anti-convulsant properties observed with these phytocannabinoids are likely elicited through a different molecular target.

The Na_V_1.7 channel is a validated target in pain research, and Na_V_1.7 inhibitors are analgesic compounds (Goodwin and McMahon, 2021). GOF mutations in the *SCN9A* gene, that result in hyperexcitable Na_V_1.7 channels, are associated with debilitating pain conditions, such as paroxysmal extreme pain disorder (Dib-Hajj et al., 2008; Stepien et al., 2020) and familial erythromelalgia (Dib-Hajj et al., 2005). Inhibition of Na_V_1.7 channel function, shown here and by others (Ghovanloo et al., 2018; Milligan et al., 2022), suggest that CBD may have therapeutic potential in alleviate symptoms in these debilitating pain conditions. In support of this theory, CBD administered in mouse models of neuropathic pain, reduced allodynia (Abraham et al., 2020; Casey et al., 2022). Our results highlight that Na_V_1.7 inhibition could be considered as a mode of analgesic action of CBD. Interestingly, the mechanism by which CBG reduced the excitability of rat dorsal root ganglion neurons was proposed to be through inhibition of Na_V_1.7 (Ghovanloo et al., 2022). Whilst no studies have assessed whether CBGA and CBDVA have analgesic effects, given the Na_V_1.7 inhibition observed with these compounds here, our future studies could examine whether CBGA and CBDVA have analgesic effects in animal models that are mediated by Na_V_1.7.

While CBD is known to interact with a diverse range of target proteins, including 5-hydroxytryptamine 1A (5-HT_1A_) receptors, γ-aminobutyric acid type A (GABA_A_) receptors, transient receptor potential (TRP) channels, the orphan G-protein-coupled receptor 55 (GPR55), and peroxisome proliferator-activated receptors (PPARs) (Pertwee et al., 2010; Anderson et al., 2019a; Watkins, 2019), research into the effects of the minor phytocannabinoids with anti-seizure properties is still in its infancy. Here we show for the first time that CBGA and CBDVA inhibit Na_V_ channels. CBGA, like CBD, has multimodal activity: it is a GPR55 and TRPV1 antagonist, a GABA_A_ positive allosteric modulator (PAM) and a T-type calcium channel inhibitor (Anderson et al., 2021b; Mirlohi et al., 2022). The molecular pharmacology of CBDVA is poorly understood, although we have recently reported it also inhibits T-type calcium channels (Udoh et al., 2022). Much work is to be done to provide a comprehensive characterisation of the mode of action of these plant cannabinoids.

In conclusion, our data provides evidence that the understudied phytocannabinoids CBGA and CBDVA inhibit voltage-gated sodium channels, *in vitro*, through variable effects on the biophysical properties of conductance and inactivation. Further research is needed to better understand the molecular actions of these cannabis constituents to guide their potential therapeutic development.

## Data Availability

The original contributions presented in the study are included in the article/supplementary material, further inquiries can be directed to the corresponding author.

## Abbreviations

AEDs, Anti-epileptic drugs; CBCA, cannabichromenic acid; CBC, cannabichromene; CBD, cannabidiol; CBDVA, cannabidivarinic acid; CBGA, cannabigerolic acid; CBG, cannabigerol; CNS, central nervous system; DS, Dravet syndrome; IC_50_, half-maximal inhibitory concentration; 5-HT, 5-hydroxytryptamine; GABA, γ‐aminobutyric acid; GOF, gain-of-function; GPR, G-protein-coupled receptor; ipi, interpulse intervals; LGS, Lennox-Gastaut syndrome; LOF, loss-of-function; Na_V_, voltage-gated sodium; PNS, peripheral nervous system; PPARs, peroxisome proliferator-activated receptors; PTZ, pentylenetetrazol; SSFI, steady-state fast inactivation; TRP, transient receptor potential; V_0.5_ act, voltage-dependence of half-activation; V_0.5_ inact, voltage-dependence of half-inactivation.
